# Compound K14 inhibits bacterial killing and protease activity in *Dictyostelium discoideum* phagosomes

**DOI:** 10.1371/journal.pone.0309327

**Published:** 2024-08-26

**Authors:** Estelle Ifrid, Hajer Ouertatani-Sakouhi, Hiba Zein El Dine, Tania Jauslin, Gianpaolo Chiriano, Leonardo Scapozza, Otmane Lamrabet, Pierre Cosson

**Affiliations:** 1 Department of Cell Physiology and Metabolism, Faculty of Medicine, University of Geneva, Geneva, Switzerland; 2 Pharmaceutical biochemistry, School of pharmaceutical sciences, University of Geneva, Geneva, Switzerland; Texas A&M University College Station: Texas A&M University, UNITED STATES OF AMERICA

## Abstract

Phagocytic cells of the mammalian innate immune system play a critical role in protecting the body from bacterial infections. The multiple facets of this encounter (chemotaxis, phagocytosis, destruction, evasion and pathogenicity) are largely recapitulated in the phagocytic amoeba *Dictyostelium discoideum*. Here we identified a new chemical compound (K14; ZINC19168591) which inhibited intracellular destruction of ingested *K*. *pneumoniae* in *D*. *discoideum* cells. Concomitantly, K14 reduced proteolytic activity in *D*. *discoideum* phagosomes. In *kil1* KO cells, K14 lost its ability to inhibit phagosomal proteolysis and to inhibit intra-phagosomal bacterial destruction, suggesting that K14 inhibits a Kil1-dependent protease involved in bacterial destruction. These observations stress the key role that proteases play in bacterial destruction. They also reveal an unsuspected link between Kil1 and phagosomal proteases. K14 can be used in the future as a tool to probe the role of different proteases in phagosomal physiology and in the destruction of ingested bacteria.

## Introduction

Phagocytic cells ingest, kill, and destroy microorganisms. In mammals, professional phagocytic cells (neutrophils and macrophages) play a key role in protecting the body by eliminating infectious microorganisms [1]. In amoebae similar mechanisms are at play [2, 3], although the aim is rather to allow amoebae to feed on ingested microorganisms. Intracellular destruction of bacteria is a complex and still incompletely understood process. In mammals, toxic ions, reactive oxygen species, lysosomal enzymes and antibacterial peptides are thought to contribute to the killing and destruction of bacteria in phagosomes [4]. However, the relative importance and specificity of each of these mechanisms remains uncertain. The amoeba *Dictyostelium discoideum* has previously been used as a model phagocytic cell to study how phagocytic cells ingest, kill, and destroy bacteria, and how pathogenic bacteria escape killing by phagocytic cells. It was shown that when a bacterium is ingested by a *D*. *discoideum* cell, it is first killed in phagosomes (i.e. it loses the ability to grow), then the bacterial membrane is permeabilized and intrabacterial constituents are destroyed by cellular mechanisms [5]. We also identified two key gene products in the bacterial destruction process, named Kil1 [6] and Kil2 [7]. Genetic inactivation of Kil1 or Kil2 leads to a strong defect in bacterial intra-phagosomal killing and destruction. Kil1 is a Golgi sulfotransferase participating in the maturation of lysosomal enzymes. Kil2 is a P-type ATPase pumping magnesium ions into the lysosomal/phagosomal lumen. In *kil2* KO cells, protease activity in phagosomes is strongly reduced [7], but this is not the case in *kil1* KO cells [8].

In previous work we searched for compounds that stimulate destruction of bacteria in phagosomes [9]. For this, we used as a tester strain *phg1A* KO *D*. *discoideum* cells, which grow very poorly on a lawn of *K*. *pneumoniae* because they fail to destroy and digest them efficiently [8]. Phg1A was originally identified as a protein necessary for efficient adhesion, the first step of the phagocytic process [10]. It was later found that Phg1A is a protein involved in the maintenance of Golgi integrity [11]. Loss of Phg1A prevents transport of the SibA adhesion molecule to the cell surface, causing a loss of cellular adhesion [12]. It also prevents proper Golgi localization of Kil1, and this causes a defect in phagosomal destruction of bacteria in phagosomes, similar to the phenotype observed in *kil1* KO cells [6]. It was expected that some compounds stimulating intracellular destruction of ingested *K*. *pneumoniae* would restore growth of *phg1A* KO cells on *K*. *pneumoniae*. Indeed, three compounds (K1-K3), were identified, which stimulated the ability of *phg1A* KO cells to feed upon *K*. *pneumoniae* bacteria [9]. The K2 compound (5-ethyl-2’-deoxyuridine) was further shown to act primarily on *K*. *pneumoniae*, rendering the bacteria more susceptible to intracellular destruction [9]. In this initial screening we did not identify compounds which would act primarily on *D*. *dictyostelium* cells by modulating intraphagosomal killing mechanisms.

Here we used a similar strategy to screen additional libraries of chemical compounds. We report the identification and characterization of several new compounds affecting the interaction between *D*. *discoideum* and *K*. *pneumoniae*. Among them, K14 acts on *D*. *discoideum* cells by modifying the physiology of the phagocytic pathway.

## Materials and methods

### Cell and reagents

All *D*. *discoideum* cells used in this study were derived from the parental DH1-10 strain [10], referred to as wild-type (WT) for simplicity. *D*. *discoideum* mutants used in this study (*phg1A* KO, *kil1* KO, *kil2* KO, *far1* KO, *lrrkA* KO, *fspa* KO and *alyL* KO were described previously and they are listed in Table 1. *D*. *discoideum* cells were grown in HL5 medium [13] at 21°C.

**Table 1:** *D*. *discoideum* strains used in this study.

Strain name	Inactivated gene name	Dictybase number DDB_	Uniprot number	Reference
DH1-10^a^	N/A	N/A	N/A	[10]
*phg1A* KO	*phg1A*	G0267444	Q55FP0	[6]
*far1KO*	*far1/grlL*	G0281211	Q54U89	[14]
*lrrkA* KO	*lrrkA/drkD*	G0281557	Q54TM7	[14]
*kil2* KO	*kil2*	G0279183	Q54X63	[15]
*alyL* KO	*alyL*	G0286229	Q54M35	[16]
*fspA* KO	*fspA*	G0277237	Q86K54	[17]
*kil1* KO	*kil1*	G0267630	Q55GK8	[6]

^a^ referred to as wild-type (WT). All mutants listed are KO strains derived from DH1-10.

The *K*. *pneumoniae* strain used in this study is a non-pathogenic laboratory strain, recently sequenced and named KpGE [18]. For all experiments it was grown in LB (lysogeny broth) at 37°C for 16h.

We used for screening a previously described collection of 8’460 chemical compounds, composed of 6’000 commercially available Maybridge compounds, and from targeted libraries potentially enriched in kinase inhibitors (1200 compounds from Prokinase), in bacterial virulence inhibitors (1’260 compounds from HD-PBL) [19]. All compounds were dissolved in DMSO at a concentration of 10mM and aliquots were stored at -20°C. Aliquots were thawed just before each experiment and immediately thrown away after use. The identification of compounds restoring growth of *phg1A* KO *D*. *discoideum* cells on *K*. *pneumoniae* bacteria was done exactly as described previously [9]. In each well, growth of *D*. *discoideum* cells on a lawn of *K*. *pneumoniae* was scored as positive when *D*. *discoideum* cleared bacteria on at least 10% of the surface.

### Intracellular destruction of bacteria

Intracellular destruction of ingested *K*. *pneumoniae* was assessed as described previously [14]. Briefly, GFP-expressing *K*. *pneumoniae* were washed twice in phosphate buffer (PB)-sorbitol (2mM Na_2_HPO4, 14.7mM KH_2_PO_4_, 100mM sorbitol, pH 6.0) then resuspended in 1 mL PB-sorbitol. The suspension was diluted 200 times in PB-sorbitol, and 150 μL of this dilution of bacteria was added on a glass slide (μ-slide 8 well glass bottom, Ibidi GmbH). 700’000 *D*. *discoideum* cells were washed twice in PB-sorbitol, resuspended in 300 μL of PB-sorbitol, and 100 μL (230’000 cells) deposited in each well. When indicated, the K14 compound was added to the well at the indicated final concentration. Alternatively, DMSO alone (0.3%) was added to the well. The cells were allowed to settle for 5 min, then imaged every 30 sec for 2 h (Nikon Eclipse Ti2). At each time point, one picture (phase contrast and GFP fluorescence) was taken in five successive focal planes (step size 3 μm) to image the whole cell volume. ImageJ was used to analyze movies. Survival analysis of phagocytosed fluorescent bacteria was computed using the Kaplan–Meier estimator. Statistical analysis was done using GraphPad Prism (V8.1.0). Since this assay measures disappearance of bacterial fluorescence in phagosome, it is referred to as bacterial destruction [5].

### Phagocytosis and macropinocytosis

Phagocytosis and macropinocytosis were measured as described previously [10] by incubating 3 x 10^5^ *D*. *discoideum* cells for 20 min in suspension in 1 mL of PB‐Sorbitol containing 1 μL of fluorescent latex beads (1-μm-diameter Fluoresbrite YG carboxylate microspheres, Polysciences, Warrington, PA, USA) and 10 μg/mL of Alexa647-dextran (Life Technologies, Eugene, OR, USA). When indicated, K14 (30 μM), folate (1 mM) or DMSO (0.3%) was also added. Cells were then washed twice with ice cold PB-sorbitol containing 0.1% NaN_3_, and the internalized fluorescence was measured by flow cytometry (Accuri).

### Cell motility

2x10^5^ *D*. *discoideum* cells were washed once with 1mL of PB-sorbitol and resuspended in 1mL of PB-sorbitol. 100 μl of the cell suspension (2 x 10^4^ cells) was deposited in each well in a 96 well plate (Greiner Bio one; Ref. 655090) and the cells were allowed to settle for 30 min. The supernatant was removed and 100 μL of PB-sorbitol supplemented or not with 1 mM folate, 30 μM K14 or DMSO was added to each well. Cells were imaged every 15 sec for 30 min with a Nikon Eclipse Ti2 equipped with a DS-Qi2 camera. The resulting movies were analyzed with the software MetaMorph (Molecular Devices) using the “Track points” function.

### Intra-phagosomal pH and proteolysis

The proteolytic activity in phasosomes was measured as already described [14] using proteolysis-sensitive bi-fluorescent beads [20]. Three‐micrometer carboxylated silica beads (Kisker Biotech; PSI‐3.0COOH) were coupled with both a proteolysis‐sensitive probe (DQ™ Green BSA; Thermo Fisher D12050) and a proteolysis‐insensitive probe (Alexa 594 succinimidyl ester; Thermo Fisher A20004). *D*. *discoideum* cells were washed with PB-sorbitol and incubated with the beads on a glass slide (μ-slide 8 well glass bottom, Ibidi GmbH), in the presence of 30 μM K14 or 0.3% DMSO. The cells were first allowed to settle for 5 min, then imaged every 75 sec for 3 h (Nikon Eclipse Ti2). At each time point, one picture (phase contrast, GFP and m-Cherry fluorescence) was taken in five successive focal planes (step size 3 μm) to image the whole cell volume. ImageJ was used to analyze movies and to quantify the fluorescence associated with the beads.

The phagosomal pH was assessed following the same procedure, using bi-fluorescent beads coupled to a pH-sensitive probe (FITC) and a pH-insensitive probe (Alexa 594), as previously described [14]. The FITC/Alexa 594 ratio decreases when beads are exposed to the acidic pH of phagosomes.

### Immunodetection of sulfated proteins

Sulfated proteins were detected by western blot as previously described [21]. Briefly, WT and *kil1* KO cells were treated either overnight or 1 h with 0.3% DMSO or 30 μM K14. The cells were then pelleted and resuspended in reducing sample buffer [20.6% (w/v) sucrose, 100 mM Tris pH6.8, 10 mM EDTA, 0.1% (w/v) bromophenol blue, 4% (w/v) SDS, 6% (v/v) b-mercaptoethanol]. 20 μL of each sample was migrated (200 V, 30 min) in a 4–20% acrylamide gel (SurePAGE Bis-Tris, Genscript #M00655), and transferred to a nitrocellulose membrane using a dry transfer system for 7 min (iBlot gel transfer device, Invitrogen #IB1001EU). The membranes were blocked during 2 h in phosphate buffered saline (PBS) containing 0.1% (v/v) Tween 20 and 7% (w/v) milk, and washed three times for 5 min in PBS + 0.1% (v/v) Tween 20. The AJ514 antibody, a recombinant version of the previously characterized 221-242-5 monoclonal antibody [22] was used to detect sulfated lysosomal enzymes.

## Results

### Chemical alteration of the interaction of phagocytic amoebae with *K*. *pneumoniae* bacteria

**Fig 1:**
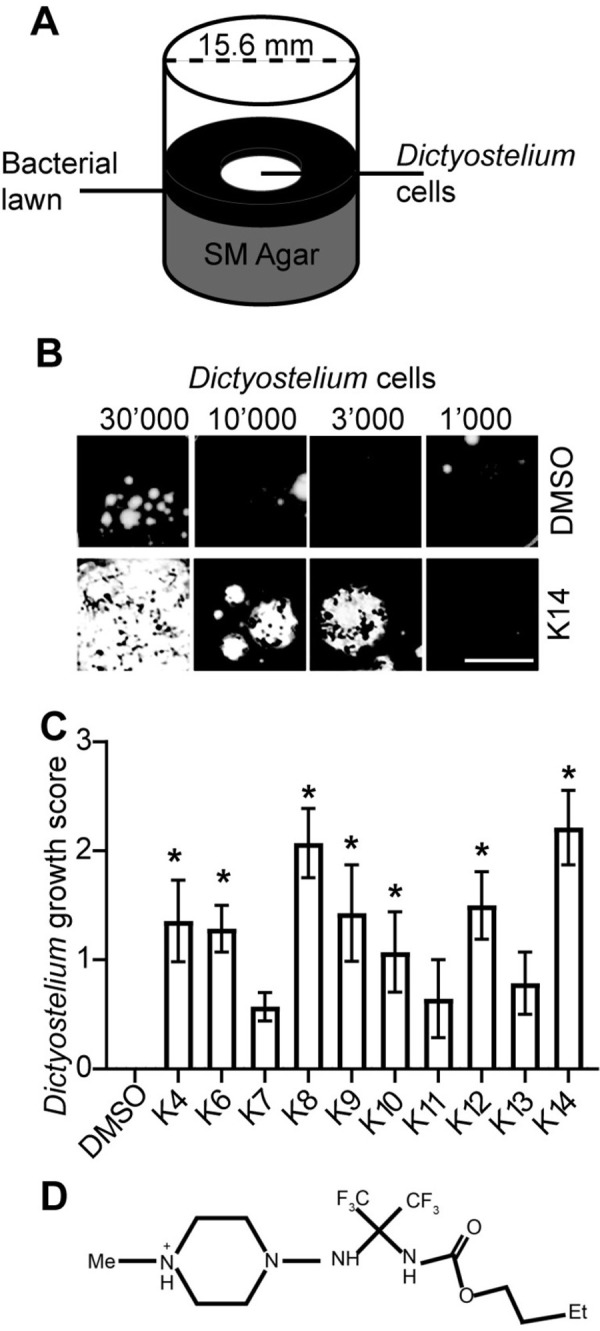
Selection of compounds restoring growth of *phg1A* KO *D*. *discoideum* cells on *K*. *pneumoniae*. **A.** *D*. *discoideum* cells were deposited on a lawn of *K*. *pneumoniae* bacteria. After 10 days, *D*. *discoideum* cells formed a phagocytic plaque if they were capable of feeding upon the bacteria. **B.** Selected compounds (here K14) increased the ability of *phg1a* KO cells to create phagocytic plaques compared with the negative control (DMSO), scale bar: 2 mm. **C.** The effect of each compound was scored from 4 (efficient growth of 1’000 *phg1A* KO cells) to 0 (no visible growth of 30’000 cells) and the score of the negative control substracted. In the example shown in B the scores would be 1 for DMSO and 3 for K14, resulting in a growth score of 3–1 = 2 for K14. Mean ± SEM; *: p<0.05; Kruskal-Wallis test, Dunn’s test. N = 7 independent experiments. **D.** Chemical structure of the K14 compound.

To identify chemical compounds which modify the interaction between *D*. *discoideum* amoebae and *K*. *pneumoniae* bacteria, we grew bacteria and amoebae in the presence of a collection of chemical compounds and identified compounds which altered the ability of amoebae to feed upon bacteria. More precisely, *K*. *pneumoniae* bacteria were deposited on nutrient agar (SM medium) in the presence of 30μM of each tested chemical compound, then 10’000 *D*. *discoideum phg1A* KO cells were added in the center of the well and allowed to grow at 21°C for 10 days (Fig 1A). In these pictures, the bacterial lawn appears black, the area from which bacteria were cleared by amoebae appear white (Fig 1B). Within cleared areas, *D*. *discoideum* cells are starved and undergo aggregation and multicellular development accounting for the small black dots present in these areas. Because they destroy ingested bacteria inefficiently, *phg1A* KO cells grew very inefficiently on a lawn of *K*. *pneumoniae*. Several libraries of chemical compounds were screened with this assay, for a total of 8’460 compounds (S1 Table in S1 File). After re-testing, 11 new hits were found to restore growth of *phg1A* KO cells on a lawn of *K*. *pneumoniae* and designated K4 to K14 (S2 Table in S1 File). All compounds were reordered except K5 which was no longer available. Their effect was then retested in a more quantitative manner, depositing on a lawn of *K*. *pneumoniae* an increasing number of *phg1A* KO *D*. *discoideum* cells, from 1’000 to 30’000 (Fig 1B), and scoring the effect of compounds on *D*. *discoideum* growth in multiple experiments. The growth of *D*. *discoideum* was increased in the presence of all compounds, and the effect was statistically significant except for K7, K11 and K13 (Fig 1C). None of the selected compounds inhibited growth of *K*. *pneumoniae* in SM medium (S1A Fig in S1 File) or in LB medium (S1B Fig in S1 File). Additional experiments were focused on the K14 compound (ZINC19168591; Fig 1D) which stimulated most effectively the ability of *phg1A* KO *D*. *discoideum* cells to feed upon *K*. *pneumoniae* bacteria. The ZINC database (zinc.docking.org) does not indicate any known or predicted activity for K14 or any interesting analog. K14 contains a cyclic piperazine structure, found in many bioactive molecules, but the significance of this observation remains unclear.

### K14 slows down intracellular destruction of bacteria in a Kil1-dependent manner

**Fig 2:**
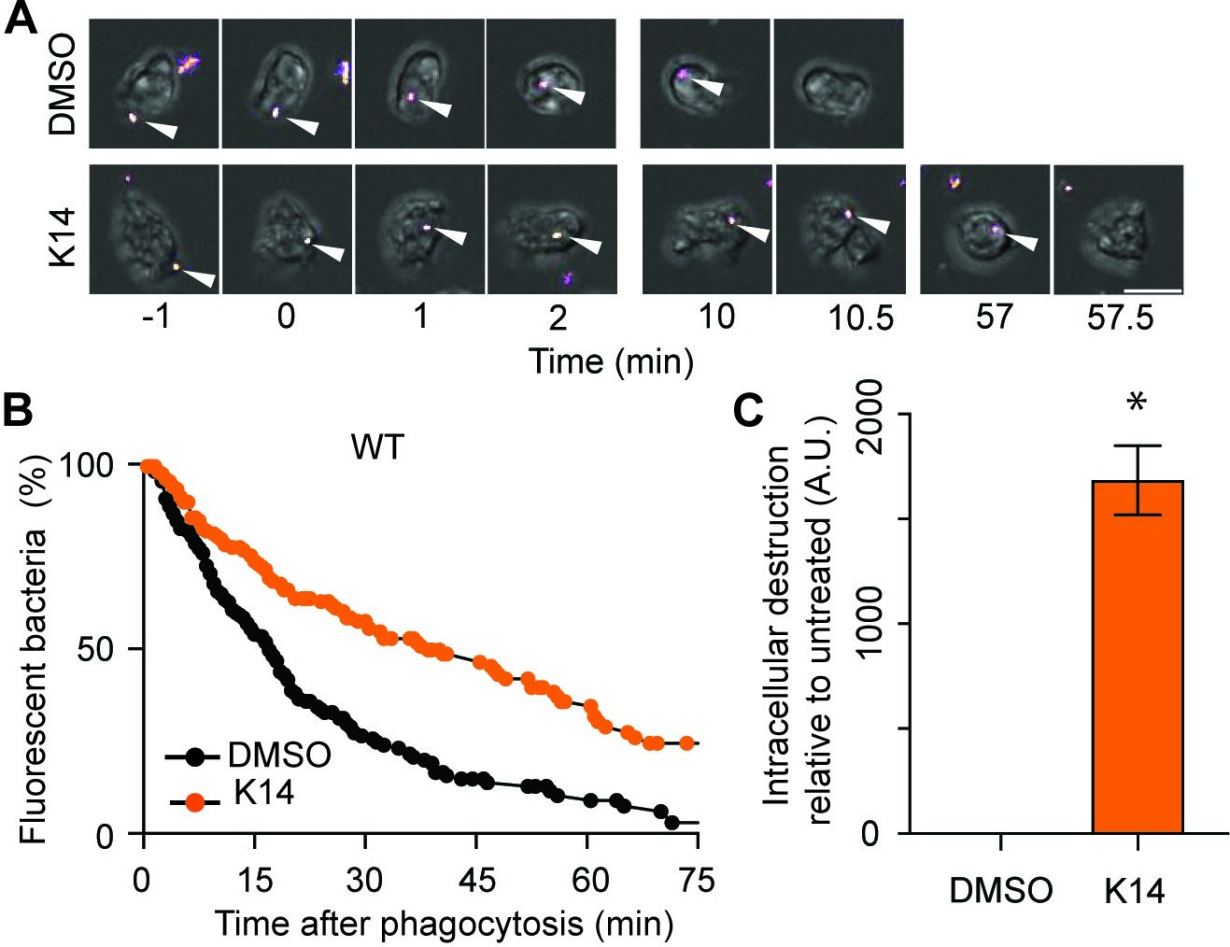
K14 inhibits the destruction of ingested *K*. *pneumoniae* in WT *D*. *discoideum* phagosomes. **A.** Time-lapse images showing a fluorescent *K*. *pneumoniae* (arrowhead) ingested by a WT *D*. *discoideum*. Extinction of the fluorescence was observed after 10 min in the upper movie (DMSO control). In the presence of K14, the fluorescence of the bacterium disappeared after 57 min (scale bar: 10 μm). **B.** Kaplan-Meier survival curves of ingested bacteria were calculated for experiments performed in the absence (DMSO) or presence of K14 (30μM) (n = 150 phagocytic events, N = 5 independent experiments). **C.** For each independent experiment, the area under each survival curve (AUC) was determined. The difference between the AUC for K14-treated and untreated cells was determined. A positive value indicated an experiment where intracellular destruction was slower than in the control. K14 slowed down significantly the intracellular destruction of *K*. *pneumoniae* by WT *D*. *discoideum* cells (mean ± SEM; *: p<0.05; Mann-Whitney test. N = 5).

The fact that K14 modifies the ability of *D*. *discoideum* to feed upon bacteria predicts that it affects the interaction between bacteria and *D*. *discoideum* cells. K14 may however act potentially on a wide variety of targets either in the bacteria or in the *D*. *discoideum* cells. To delineate better the mode of action of K14, we tested its ability to modify the kinetics of *K*. *pneumoniae* bacteria destruction in phagosomes. For this, *D*. *discoideum* cells were incubated in the presence of GFP-expressing *K*. *pneumoniae* and imaged every 30 seconds. Bacteria were ingested, and the disappearance of their fluorescence indicated the time when they were destroyed in the phagosome (Fig 2A). In WT *D*. *discoideum* cells, ingested *K*. *pneumoniae* were rapidly destroyed. However, K14 slowed down markedly intracellular destruction of ingested bacteria in WT *D*. *discoideum* phagosomes (Fig 2B). This effect was visible in multiple experiments and was statistically significant (Fig 2C).

**Fig 3:**
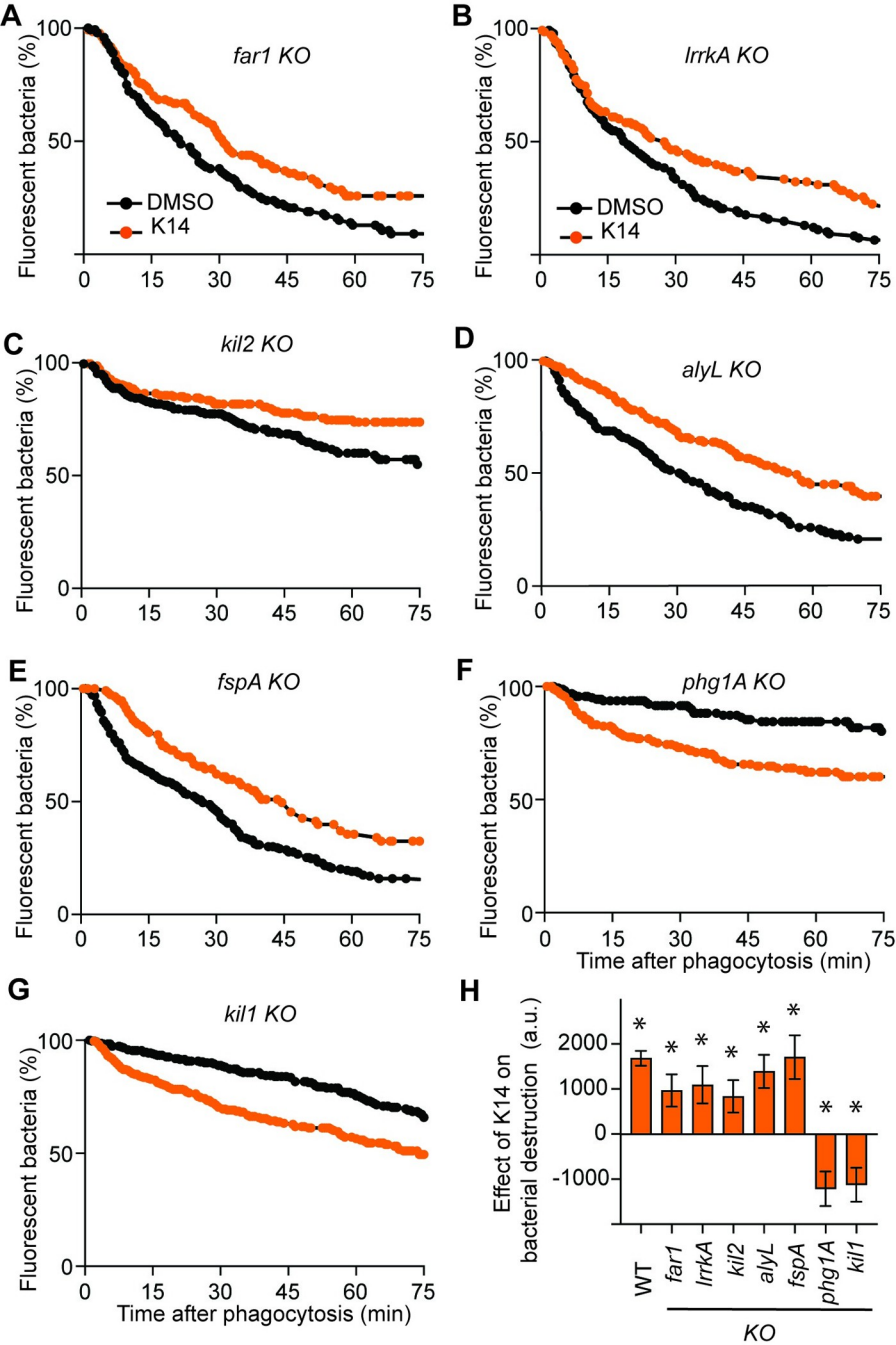
Effect of K14 on intracellular destruction of *K*. *pneumoniae* in *D*. *discoideum* mutants. The effect of K14 (30μM) on intracellular destruction of ingested *K*. *pneumoniae* bacteria was determined as described in the legend to Fig 2 in *far1* KO (**A**), *lrrkA* KO (**B**), *kil2* KO (**C**), *alyL* KO (**D**), *fspA* KO (**E**), *phg1A* KO (**F**) and *kil1* KO cells (**G**). The destruction of ingested *K*. *pneumoniae* is shown in each mutant cell treated with K14 (orange), or mock-treated with only DMSO (black). **H**. For each individual experiment the effect of K14 was assessed by measuring the area under the curve in untreated vs K14-treated cells (mean ± SEM; *: p<0.05; Mann-Whitney-test, *far1* KO, *phg1A* KO: N = 6; WT, *lrrkA* KO: N = 5; *kil2* KO, *kil1* KO: N = 11; *fspA KO*: N = 4; *alyL* KO: N = 7 independent experiments).

In principle, if K14 inhibits a gene product necessary for efficient destruction (e.g. Kil2), its effect should be lost in the corresponding knockout strain (e.g. *kil2 KO* strain). To better delineate the molecular process inhibited by K14, we tested the effect of K14 on a collection of *D*. *discoideum* mutants known to exhibit slow intracellular bacterial destruction. K14 inhibited bacterial destruction in *far1* KO, *lrrkA* KO, *kil2* KO, *alyL* KO and *fspA* KO cells (Fig 3A–3E), suggesting that K14 inhibits a cellular process still functional in these mutant strains. On the contrary, in *phg1A* KO cells bacterial destruction was not slowed down by K14, but rather significantly accelerated (Fig 3F). Previous experiments have shown that *phg1A* KO cells destroy poorly ingested bacteria largely because they are depleted in the Kil1 protein [8]. Remarkably, like in *phg1A* KO cells, in *kil1* KO cells K14 did not slow down bacterial destruction but rather accelerated it (Fig 3G). These observations indicate that the inhibitory effect of K14 is exerted on a target which is inactive in *phg1A* and *kil1* KO cells. Together these results indicate that K14 inhibits intracellular destruction of bacteria in a Phg1/Kil1-dependent manner. In the absence of this dominant Kil1-dependent inhibition, a weaker stimulatory effect of K14 is revealed. This weaker effect presumably accounts for the fact that K14 stimulates growth of *phg1A* KO cells on *K*. *pneumoniae* bacteria.

**Fig 4:**
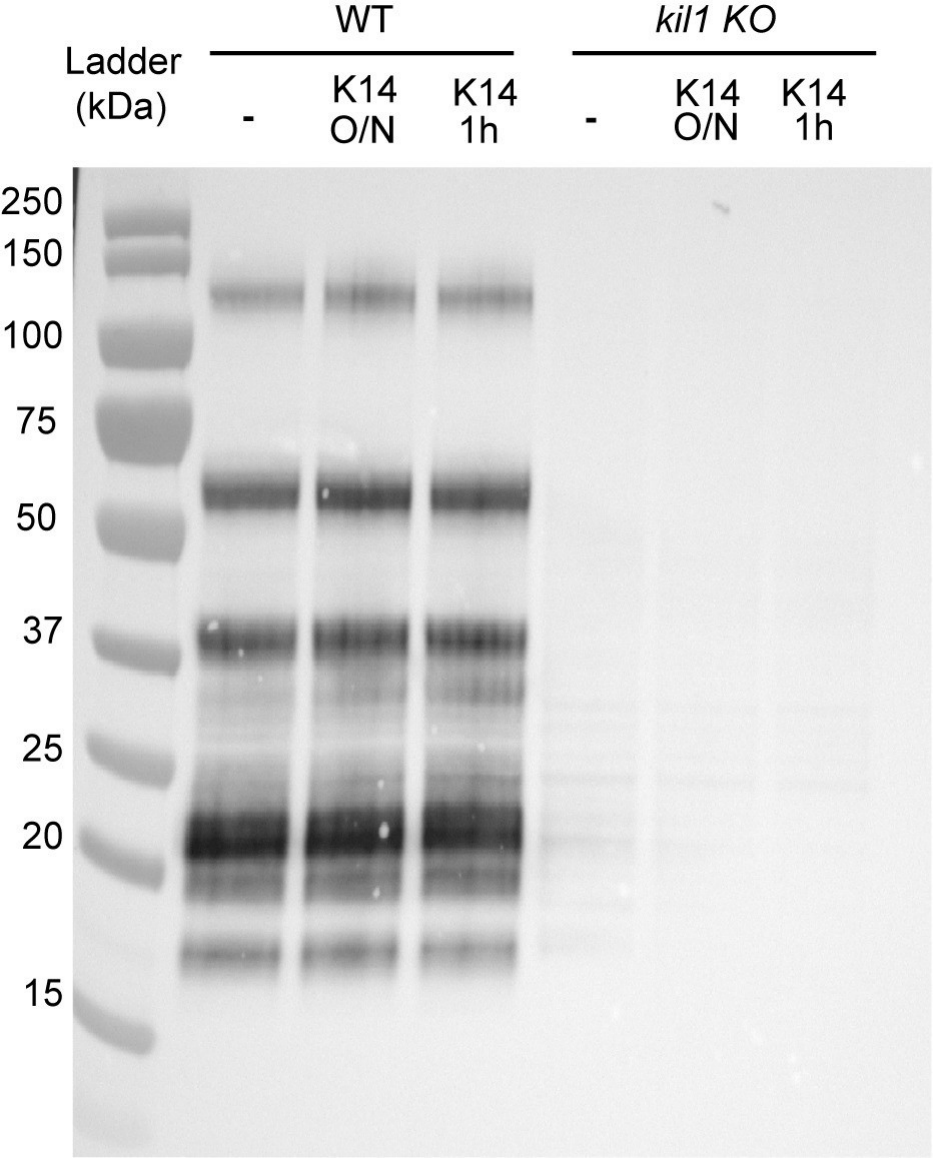
The sulfation of proteins is not affected by a K14 treatment. The AJ514 scFv antibodies recognizes the sulfated common antigen 1 and was used to detect sulfated proteins by western blot. Many proteins were detected in lysates from WT *D*. *discoideum* cells but not in the sulfation-defective *kil1* KO mutant. Incubation of the cells with K14 (30μM) for 1h or overnight prior to lysis did not modify the sulfation pattern observed. The original uncropped gel is shown in S4 Fig in S1 File.

As detailed above, K14 could inhibit bacterial destruction by inhibiting Phg1, Kil1, or one or several gene products dependent on Kil1 (e.g. proteins sulfated by Kil1). If K14 inhibited Phg1, it should induce a phenotype similar to that of *phg1A* KO cells, i.e. inhibit bacterial destruction but also cellular adhesion and phagocytosis [10]. K14 did not inhibit phagocytosis (S2 Fig in S1 File), indicating that the primary target of K14 is not Phg1 itself. To test whether K14 inhibited Kil1 itself, we tested whether sulfation of proteins was modified by K14 in WT or *kil1* KO cells. For this we used an antibody against sulfated antigens (ABCD_AJ514) to assess the presence of sulfated proteins in cell lysates. As expected, many sulfated proteins were detected in WT cells, and virtually none in *kil1* KO cells (Fig 4). WT or *kil1* KO cells were treated with K14 for either 1 h or 16 h (overnight), and this did not modify the number of sulfated proteins detected in cellular lysates, nor the intensity of the observed signal (Fig 4). These results indicate that K14 does not act by directly inhibiting Kil1 sulfation activity, but rather by interfering with a Kil1-dependent process, most likely inhibiting one or several Kil1-dependent lysosomal enzyme. This interpretation is also coherent with the fact that a treatment with K14 inhibits bacterial destruction much more efficiently than loss of Kil1 (in *kil1* KO cells).

**Fig 5:**
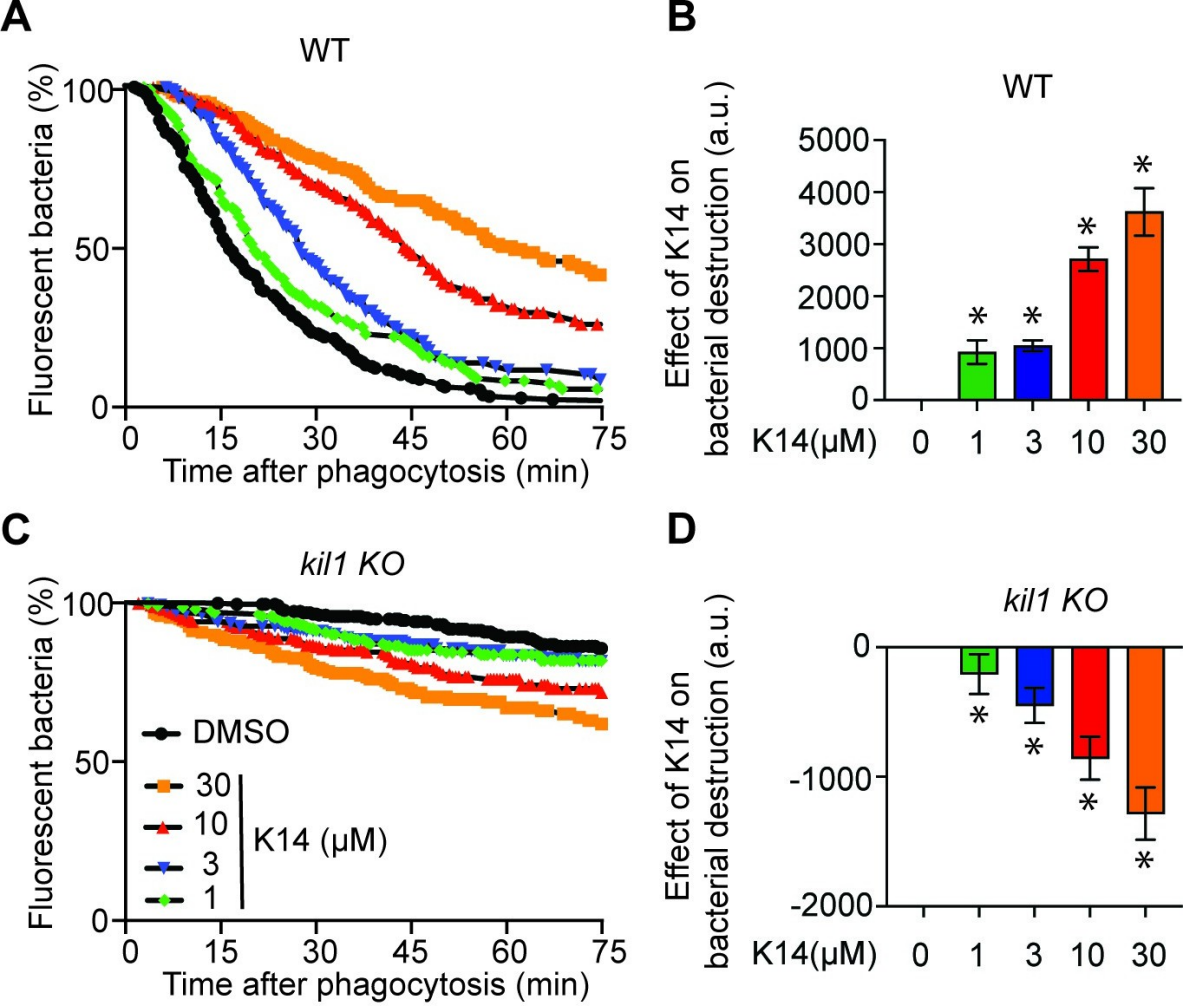
K14 inhibits intracellular destruction of *K*. *pneumoniae* in WT cells and stimulates it in *kil1* KO cells at similar concentrations. **A.** The effect of K14 on the intracellular destruction of *K*. *pneumoniae* by WT *D*. *discoideum* cells was determined as described in the legend to Fig 2. **B.** K14 inhibited significantly bacterial destruction at concentrations of 1μM and higher (mean ± SEM; *: p<0.05; Mann-Whitney test, N = 5 independent experiments). **C**. In *kil1* KO cells, intracellular destruction of ingested bacteria was much slower than in WT cells, and it was stimulated by K14 at a concentration of 1μM and higher. **D.** Analysis of 7 independent experiments revealed that the stimulatory effect of K14 was statistically significant (mean ± SEM; *: p<0.05; Mann-Whitney test, N = 7).

All the experiments described above were carried out using a high concentration of K14 (30μM). We next tested the effect of decreasing K14 concentrations. K14 inhibited significantly bacterial destruction in WT cells at a concentration of 1μM, and the inhibition was more prominent at higher concentrations (Fig 5A and 5B). Similarly, K14 stimulated significantly destruction of *K*. *pneumoniae* in *kil1* KO cells at a concentration of 1μM and the stimulatory effect increased at higher concentrations (Fig 5C and 5D). These observations confirm that both the inhibitory and stimulatory effects of K14 are dose-dependent as would be expected from a classical small molecule inhibitor/activator.

### K14 inhibits phagosomal proteolysis in WT but not in kil1 KO cells

**Fig 6:**
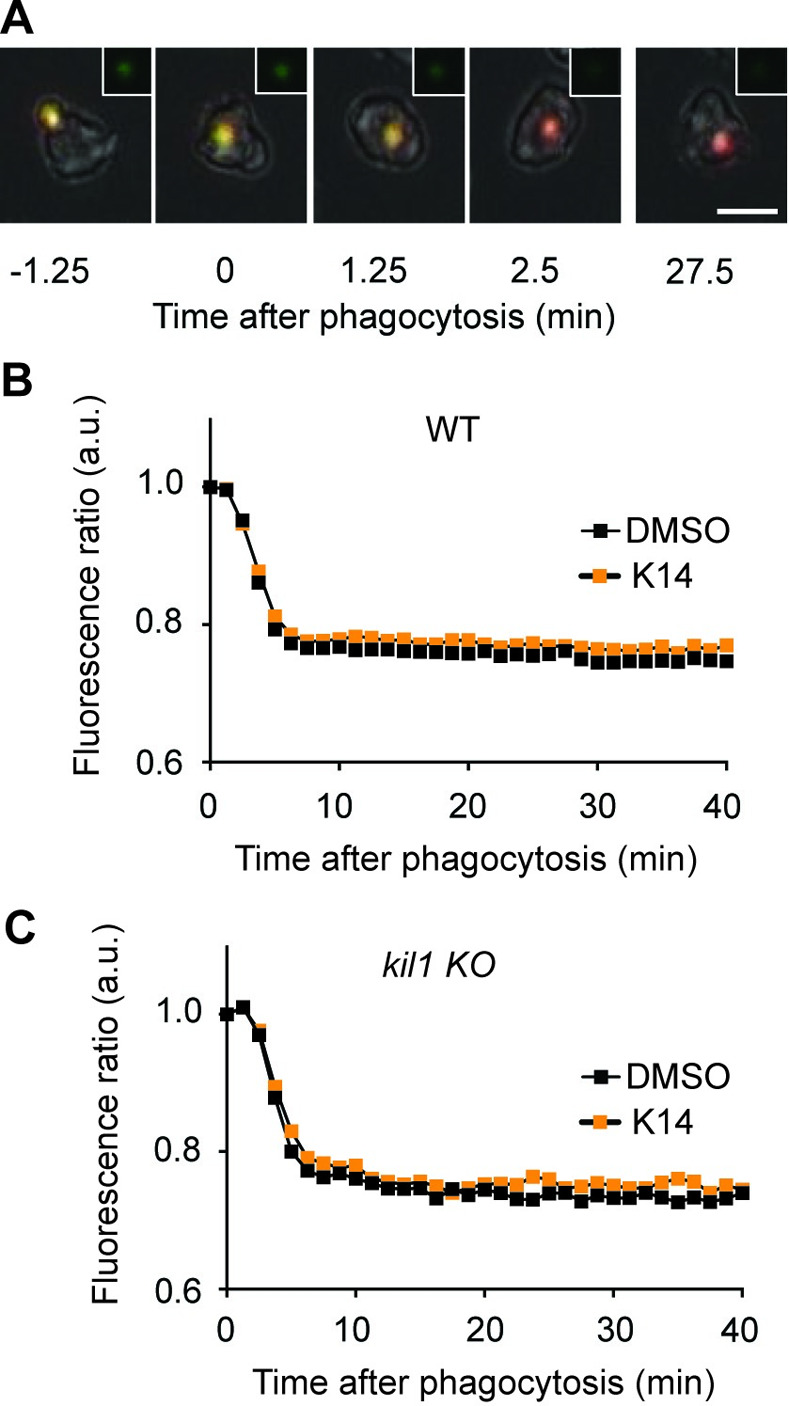
K14 does not alter the acidification of phagosomes. **A.** Acidification of phagosomes was assessed by measuring the fluorescence of ingested silica beads coupled to two fluorophores, one quenched by pH (FITC, green), one not (Alexa 594, red). Shortly after ingestion (t = 0), FITC fluorescence was quenched by the acidic phagosomal pH and the fluorescence of the beads switched from yellow (green+red) to red. For each picture an inset shows the fluorescence of the bead in the FITC channel. Scale bar: 10μm. **B.** Acidification of phagosomes proceeded with the same kinetics in WT cells treated with K14 (30μM) or not (DMSO). **C.** Similarly, in *kil1 KO* cells, acidification of phagosomes was indistinguishable from acidification in WT cells and was not inhibited by K14. (mean ± SEM; WT: N = 13; *kil1* KO: N = 11).

The fact that K14 exhibits a different effect on different *D*. *discoideum* mutant strains suggests that it acts on the *D*. *discoideum* cells rather than on the bacteria, although other more complex interpretations are also possible. More precisely, K14 may modify the physiology of the phagocytic pathway, since it alters intraphagosomal destruction of bacteria. To test this hypothesis further, we assessed the effect of K14 directly on *D*. *discoideum* cells, in the absence of bacteria. Exposure to K14 did not modify phagocytosis or macropinocytosis rates in *D*. *discoideum* cells (S2 Fig in S1 File), nor did it alter cell motility (S3 Fig in S1 File). In both experiments, folate was used as a positive control, since it was previously shown to stimulate cell motility, macropinocytosis and phagocytosis [17] (S2 and S3 Figs in S1 File). We also measured the acidification of phagosomes using beads coated with two fluorophores, one pH-sensitive (FITC) and one not (Alexa 594). Rapid acidification of phagosomes is revealed by the quenching of the FITC fluorescence (Fig 6A). We measured the decrease in the FITC/Alexa 594 fluorescence ratio following ingestion of individual beads, and observed that K14 does not modify the kinetics of acidification of newly formed phagosomes in WT *D*. *discoideum* cells (Fig 6B) or in *kil1* KO cells (Fig 6C). Overall, these results established that K14 does not profoundly affect the physiology of the phagocytic pathway in *D*. *discoideum*.

**Fig 7:**
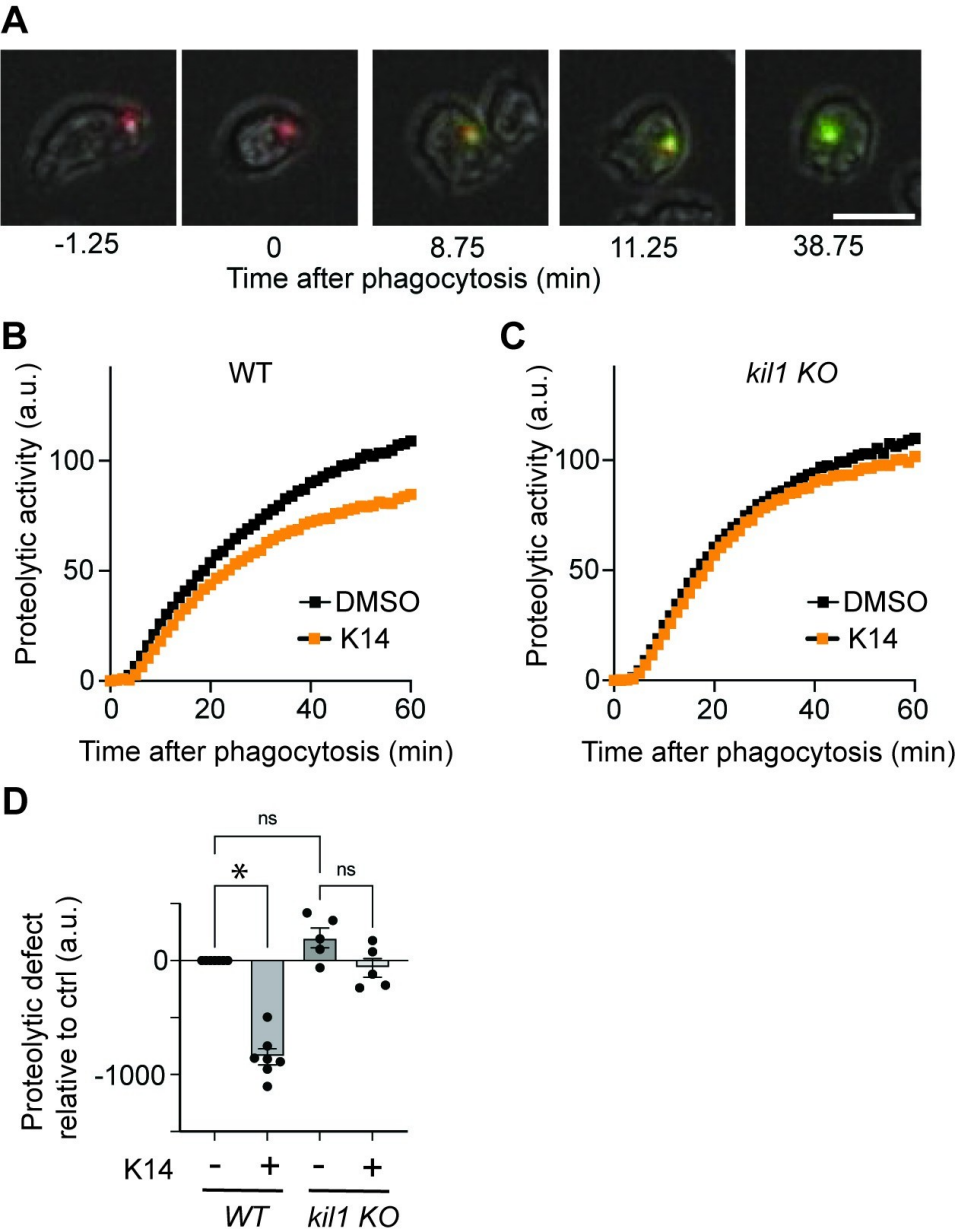
K14 inhibits proteolytic activity in phagosomes in WT but not in *kil1* KO cells. **A.** Proteolytic activity in phagosomes was assessed by measuring the fluorescence of ingested silica beads coupled to BSA-DQ Green. Following ingestion (t = 0), digestion of BSA and release of DQ Green leads to a marked increase in fluorescence. Beads were also coupled to a proteolysis-insensitive fluorophore to allow continuous observation (Alexa 594, red). Scale bar: 10μm. **B.** Release of fluorescent DQ-Green in phagosomes was measured as a function of time to assess the proteolytic activity in maturing phagosomes. Phagosomal proteolysis was inhibited in WT *D*. *discoideum* cells treated with K14 (30μM). (N = 5 independent experiments, n = 75 ingested beads). **C.** Proteolytic activity in phagosomes was not modified in *kil1* KO cells exposed to K14 compared with DMSO. (N = 7 independent experiments, n = 105 ingested beads). **D.** To evaluate the statistical validity of these results, the results of individual experiments were compared (mean ± SEM; *: p<0.05; Mann-Whitney test).

We then measured the proteolytic activity in phagosomes of cells exposed to K14. For this, we used silica beads coupled to two fluorophores: DQ Green coupled to BSA and Alexa 594 [20]. The fluorescence of DQ Green is quenched when it is coupled to BSA, but it increases sharply when proteolysis of BSA releases DQ Green from the beads. On the contrary, Alexa 594 provides a constant signal, insensitive to phagosomal proteolysis. As previously reported [14], proteolysis is detectable within minutes following ingestion of beads (Fig 7A). The released DQ Green fluorescence increases steadily and reaches a plateau after approximately 30 minutes (Fig 7B). In WT cells, addition of K14 significantly inhibited the proteolysis (Fig 7B and 7D). On the contrary, in *kil1* KO cells, K14 did not inhibit proteolysis (Fig 7C and 7D). These results suggest that K14 inhibits destruction of *K*. *pneumoniae* bacteria in WT cells, at least in part, by inhibiting the activity of proteases in phagosomes. On the contrary, in *kil1* KO cells, K14 does not inhibit proteolysis, and fails to inhibit bacterial destruction.

## Discussion

In this study, we identified K14 as a compound modulating the intracellular destruction of *K*. *pneumoniae* bacteria in *D*. *discoideum* phagosomes. In WT cells, K14 inhibits destruction of *K*. *pneumoniae* bacteria in phagosomes. Two separate lines of evidence indicate that this effect is primarily due to the action of K14 on cellular physiology. First K14 inhibits destruction of ingested bacteria in the phagosomes of WT cells, but not in *phg1A* KO or *kil1* KO cells. This observation suggests that K14 acts on a *D*. *discoideum* cellular mechanism which is inactive in *kil1* KO and in *phg1A* KO cells. K14 does not modify protein sulfation by Kil1, but probably acts on a lysosomal enzyme which requires sulfation by Kil1 to be active. Second, in the absence of bacteria, the enzymatic activity of proteases in WT *D*. *discoideum* phagosomes is inhibited by K14. Remarkably, in *kil1* KO cells, K14 does not inhibit proteolytic activity in phagosomes, as would be expected if K14 inhibits a Kil1-dependent phagosomal protease. This observation suggests that K14 inactivates a phagosomal protease which is inactive or depleted from phagosomes in *kil1* KO cells. The inhibition of bacterial destruction and of phagosomal protease activity is observed within minutes of exposure to K14. The effect of K14 may thus be achieved either by modifying the composition of newly forming phagosomes (e.g. by inhibiting the phagosomal targeting of a critical protease), or by directly inhibiting the activity of a protease.

If phagosomes of WT cells contain a protease inhibited by K14, and this protease is absent or inactive in *kil1* KO cells, one would expect the total proteolytic activity to be reduced in *kil1* KO cells. Previous analysis, as well as this study indicate on the contrary that the total proteolytic activity in *kil1* KO cells is quantitatively similar to that in WT cells. This suggests that in *kil1* KO cells the loss of activity of a K14-dependent protease is compensated by the up-regulation of some other K14-insensitive protease. This compensatory effect explains why previous studies failed to detect an alteration of phagocytic proteases in *kil1* KO cells. It should be emphasized that the tools that we used in this study to detect phagosomal protease activity detect any protease capable of digesting BSA. More specific substrates may be necessary to detect the modification of protease activities in *kil1* KO cells compared to WT cells.

In *phg1A* KO cells, as well as in *kil1* KO cells, K14 does not inhibit intracellular destruction of bacteria, and on the contrary it stimulates it. It is likely that this stimulation of bacterial destruction accounts for the increased growth of *phg1A* KO cells on a lawn of *K*. *pneumoniae* bacteria, the phenotype initially used to identify K14. In retrospect, it is curious to note that the K14 compound was not identified based on its primary inhibitory effect on WT *D*. *discoideum* cells, but rather on a weaker stimulatory effect observed only in *phg1A* KO and *kil1* KO mutant cells. While it may be interesting to investigate the molecular nature of this secondary stimulatory effect of K14, this effect is quantitatively less pronounced than the primary inhibitory effect of K14.

**Fig 8:**
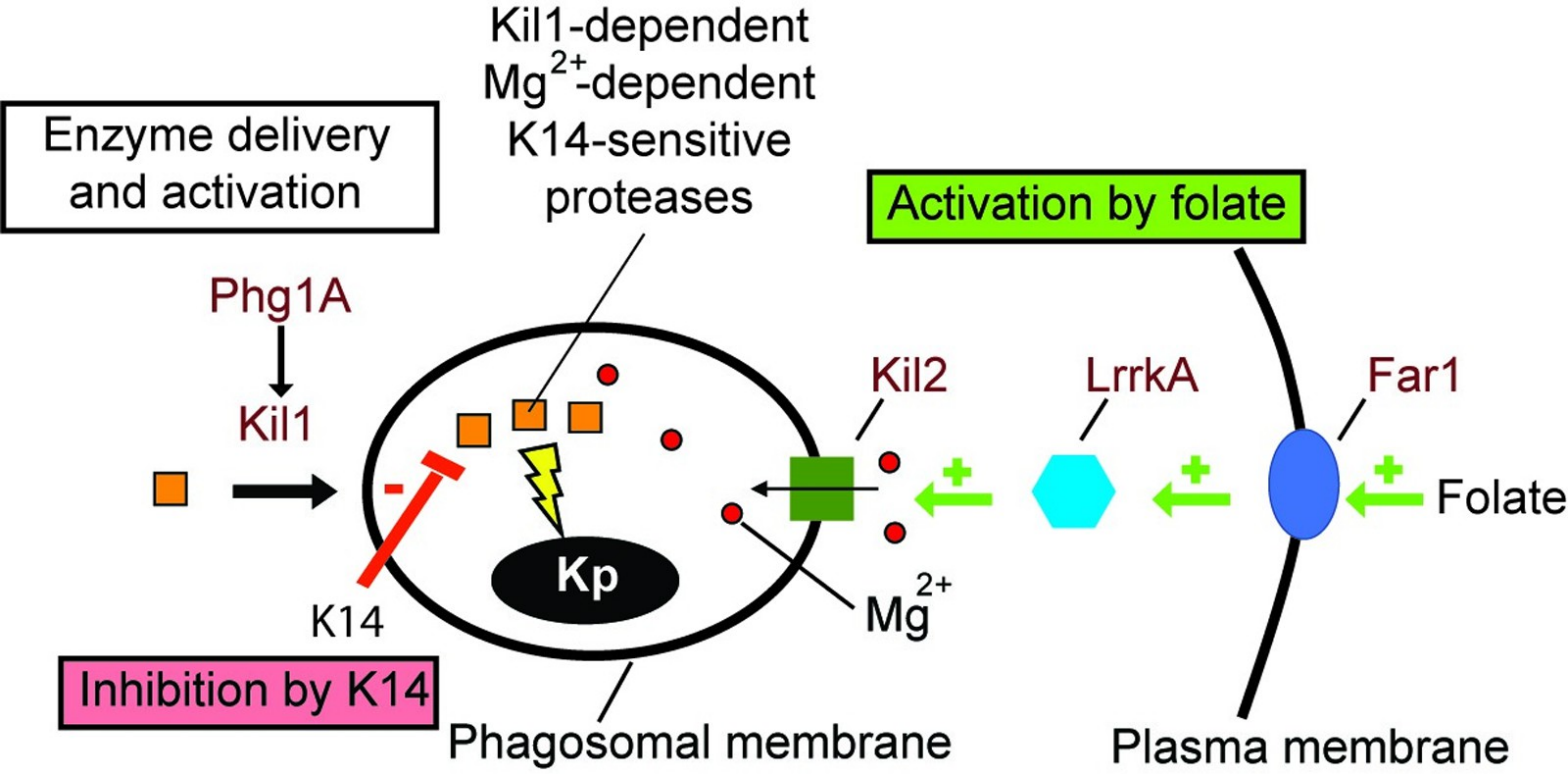
Effects of folate and K14 on phagosomal physiology. The primary effect of folate is to stimulate the Far1-LrrkA-Kil2 pathway, ultimately increasing magnesium concentration in phagosomes. Magnesium stimulates phagosomal proteases necessary for destruction of *K*. *pneumoniae* (Kp) bacteria. The primary effect of K14 is to inhibit Kil1-dependent proteases involved in bacterial destruction. Phagosomal proteases involved in bacterial destruction are stimulated by magnesium, inhibited by K14, and by genetic inactivation of Kil1.

K14 is the second chemical compound identified in our studies that modulates the physiology of phagosomes and destruction of bacteria (schematized in Fig 8). Indeed, previous studies showed that folate, a bacterially secreted compound, stimulates destruction of *K*. *pneumoniae* in phagosomes. This stimulatory effect was lost in mutants where folate sensing was lost: *far1* KO (the surface receptor for folate), *lrrkA* KO (a cytosolic kinase), and *kil2* KO (an ion pump believed to pump magnesium into the phagosome lumen). In these mutants, a decrease in phagosomal proteolytic activity accompanied, and probably caused the slower destruction of *K*. *pneumoniae* bacteria [14]. Folate was proposed to stimulate the Far1-LrrkA-Kil2 pathway, inducing an increase in phagosomal magnesium and thus stimulating phagosomal proteases and the destruction of ingested bacteria. In the current study, K14 slows down bacterial destruction by inhibiting the activity or the delivery of a Kil1-dependent protease in phagosomes. These new observations stress further the importance of proteolytic activity in *D*. *discoideum* phagosomes to achieve efficient destruction of bacteria. To date, no individual phagosomal protease has been identified that is required for efficient destruction of bacteria in phagosomes. A partially purified extract of *D*. *discoideum* exhibiting bacteriolytic activity contained 15 proteases [23] (tpp1B, C, E, F; ctsB, D, Z; cprA, D, E, G; Q54CF7, Q54MN6, Q54VR1, Q54F16/p34), at least two of which (ctsD, p34) were previously shown to be present in the endocytic/phagocytic pathway [24, 25]. Our results may help in the identification of a phagosomal protease involved in the destruction of *K*. *pneumoniae* bacteria: this protease(s) should be inactive (or absent from phagosomes) in *kil1* KO cells, stimulated by magnesium ions, and inhibited (or depleted from phagosomes) in cells exposed to K14.

## Supporting information

S1 File Supporting information. (PDF)
