# Supplementary material for: Compound K14 inhibits bacterial killing and protease activity in Dictyostelium discoideum phagosomes
Source: PLoS One. 2024 Aug 26;19(8):e0309327. doi: 10.1371/journal.pone.0309327 (PMC11346726; doi:10.1371/journal.pone.0309327)
Supplement: S1 File — (PDF) [file pone.0309327.s001.pdf]

Supporting information

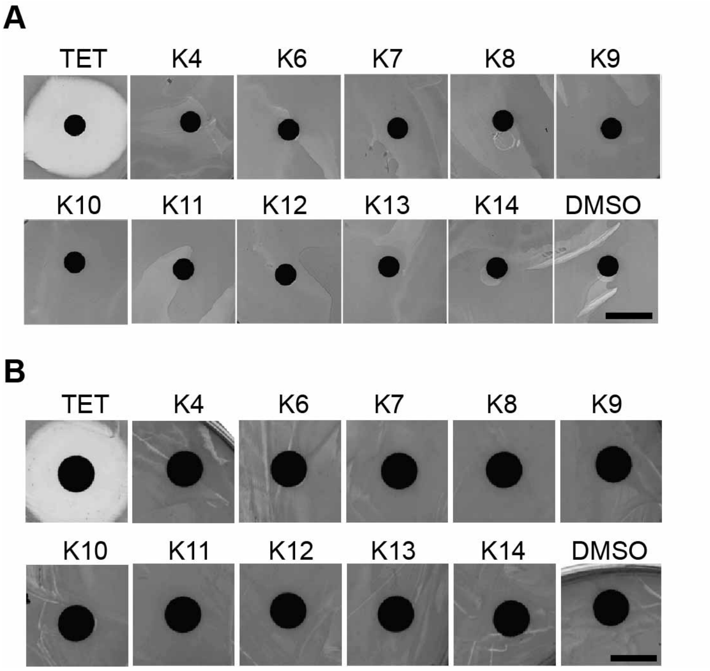

S1 Fig. Selected compounds exhibit no antibiotic activity against *K. pneumoniae*. *K. pneumoniae* bacteria were plated on LB**-** (**A**) or SM- **(B)** agar plates. All compounds were dissolved in DMSO at a concentration of 10mM. Paper discs with 20 µl each stock compound, the positive control (tetracycline, TET) or the negative control (DMSO) were then placed on the plates and the bacteria allowed to grow at 37°C overnight. After an overnight incubation at 37°C, a halo of bacterial growth inhibition was observed around the disk containing tetracycline, but none of the selected compounds showed a similar effect (scale bar: 7 mm).

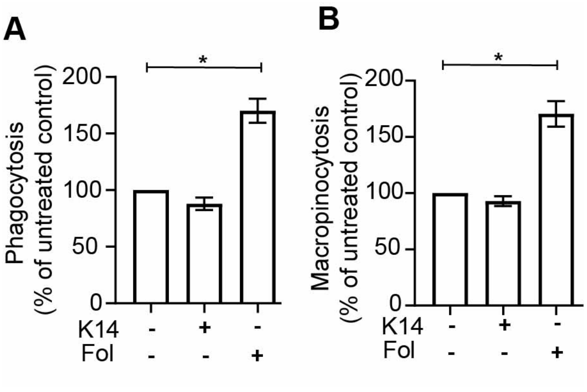

S2 Fig. Folate stimulates phagocytosis and macropinocytosis in *D. discoideum*, K14 does not. WT *D. discoideum* cells were incubated for 20 min in PB-sorbitol in the presence of fluorescent latex beads and dextran. Cells were then washed and phagocytosis of latex beads was determined by flow cytometry (**A**), as well as macropinocytosis of dextran (**B**). Folate, but not K14, stimulated phagocytosis and macropinocytosis in WT cells (mean ± SEM; *: p<0.05; Kruskal-Wallis test, Dunn's test. N= 8 independent experiments).

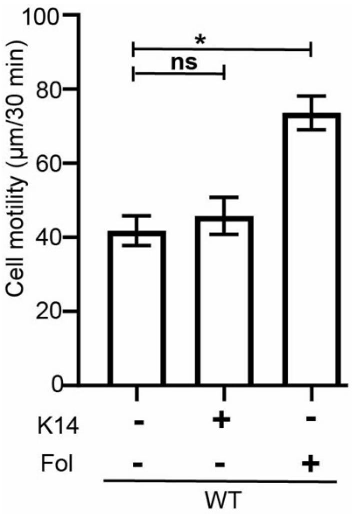

S3 Fig. The motility of WT *D. discoideum* is increased by folate but not by K14. WT *D. discoideum* cells were deposited on a glass surface in PB-sorbitol and their motility assessed for 30 min in the absence or presence of folate or K14 (mean ± SEM; *: p<0.05; Kruskal-Wallis test, Dunn's test. N=8).

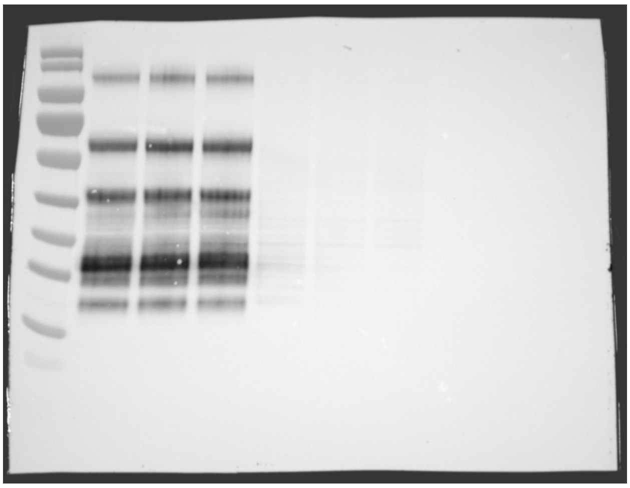

S4 Fig. Original gel shown in Fig 4.

S1 Table. Number and origin of selected compounds.

Library	Compounds	Primary Hits	Confirmed Hits	Hit rate (%)
HD-PBL	1260	9	2	0.15
Prokinase	1200	0	0	0
Maybridge	6000	50	9	0.15
**Total**	**8460**	**59**	**11**	**0.3**

S2 Table. Description of 11 confirmed hits that restore growth of *phg1A* KO cells on a lawn of *K. pneumoniae* bacteria.

Code	Reference	Mw	Source
K4	ZINC05717049	309.27	HD-PBL
K5	ZINC05332174	217.31	HD-PBL
K6	ZINC00066194	262.32	Maybridge
K7	ZINC01044636	387.21	Maybridge
K8	ZINC08616759	475.17	Maybridge
K9	ZINC4324614	376.41	Maybridge
K10	ZINC19834843	364.84	Maybridge
K11	ZINC00153843	226.23	Maybridge
K12	ZINC54379044	279.33	Maybridge
K13	ZINC00172866	330.72	Maybridge
K14	ZINC19168591	380.32	Maybridge
